# Candida albicans Augments Staphylococcus aureus Virulence by Engaging the Staphylococcal *agr* Quorum Sensing System

**DOI:** 10.1128/mBio.00910-19

**Published:** 2019-06-04

**Authors:** Olivia A. Todd, Paul L. Fidel, Janette M. Harro, Jamese J. Hilliard, Christine Tkaczyk, Bret R. Sellman, Mairi C. Noverr, Brian M. Peters

**Affiliations:** a Integrated Program in Biomedical Sciences, College of Graduate Health Sciences, University of Tennessee Health Science Center, Memphis, Tennessee, USA; b Department of Oral Biology, School of Dentistry, Louisiana State University Health Sciences Center, New Orleans, Louisiana, USA; c Department of Microbiology, Immunology, & Parasitology, School of Medicine, Louisiana State University Health Sciences Center, New Orleans, Louisiana, USA; d Department of Microbial Pathogenesis, School of Dentistry, University of Maryland—Baltimore, Baltimore, Maryland, USA; e Department of Microbial Sciences, AstraZeneca, Gaithersburg, Maryland, USA; f Department of Microbiology and Immunology, Tulane University School of Medicine, New Orleans, Louisiana, USA; g Department of Clinical Pharmacy and Translational Science, College of Pharmacy, University of Tennessee Health Science Center, Memphis, Tennessee, USA; h Department of Microbiology, Immunology, and Biochemistry, College of Medicine, University of Tennessee Health Science Center, Memphis, Tennessee, USA; University of Texas Health Science Center

**Keywords:** Candida albicans, intraabdominal infection, polymicrobial

## Abstract

Candida albicans and Staphylococcus aureus are among the most prevalent nosocomial pathogens that are responsible for severe morbidity and mortality, even with appropriate treatment. Using a murine model of polymicrobial intra-abdominal infection (IAI), we have previously shown that coinfection with these pathogens results in synergistic lethality that is partially dependent on exacerbated prostaglandin signaling, while monomicrobial infection is nonlethal. Therefore, the objective of this study was to identify staphylococcal virulence determinants that drive lethal synergism during polymicrobial IAI. Using the toxigenic S. aureus strain JE2, we observed that coinfection with C. albicans led to a striking 80 to 100% mortality rate within 20 h postinoculation (p.i.) while monomicrobial infections were nonlethal. Use of a green fluorescent protein (GFP)-P3 promoter S. aureus reporter strain revealed enhanced activation of the staphylococcal *agr* quorum sensing system during *in vitro* polymicrobial versus monomicrobial growth. Analyses by quantitative real-time PCR (qPCR), Western blot, and toxin functional assays confirmed enhanced *agr*-associated gene transcription and increases in secreted alpha- and delta-toxins. C. albicans-mediated elevated toxin production and hemolytic activity were determined to be *agrA* dependent, and genetic knockout and complementation of *hla* identified alpha-toxin as the key staphylococcal virulence factor driving lethal synergism. Analysis of mono- and polymicrobial infections 8 h p.i. demonstrated equivalent bacterial burdens in the peritoneal cavity but significantly elevated levels of alpha-toxin (3-fold) and the eicosanoid prostaglandin E_2_ (PGE_2_) (4-fold) during coinfection. Importantly, prophylactic passive immunization using the monoclonal anti-alpha-toxin antibody MEDI4893* led to significantly improved survival rates compared to those following treatment with isotype control antibody. Collectively, these results define alpha-toxin as an essential virulence determinant during C. albicans-S. aureus IAI and describe a novel mechanism by which a human-pathogenic fungus can augment the virulence of a highly pathogenic bacterium *in vivo*.

## Abstract (precis)

Relatively little is known about the complex interactions and signaling events that occur between microbes and even less so about how microbial “cross talk” shapes human health and disease. Candida albicans (a fungus) and Staphylococcus aureus (a bacterium) are formidable human nosocomial pathogens, causing severe morbidity and mortality. Moreover, they are frequently coisolated from central venous catheters and deep-seated infections, including intra-abdominal sepsis. In this work, we have shown that coinfection with C. albicans and S. aureus is highly lethal, leading to >80% mortality by day 1 postinfection, whereas monoinfection with C. albicans or S. aureus does not cause mortality. This infectious synergism is dependent on the expression of staphylococcal alpha-toxin, and secretion of this potent virulence factor is actually augmented by C. albicans via an *agr*-dependent mechanism. Moreover, prophylactic neutralization of alpha-toxin with a monoclonal antibody is sufficient to elicit protection during coinfection. Therefore, we have demonstrated that a pathogenic fungus can enhance virulence determinants of a bacterium *in vivo* with devastating consequences to the host. These results have important implications in the surveillance and treatment of polymicrobial disease and highlight the dynamic intersection of environment, pathogens, and host.

## INTRODUCTION

The pathogenic fungus Candida albicans and the ubiquitous bacterial pathogen methicillin-resistant Staphylococcus aureus (MRSA) remain serious clinical threats (1, 2). Together, these microorganisms rank among the most prevalent causes of nosocomial sepsis and catheter-related bloodstream infections, and recent reports have identified their coisolation with increasing frequency (3, 4). While polymicrobial infection is often associated with poor patient prognosis, studies designed to mechanistically evaluate microbial community composition and fungal-bacterial interactions in the context of host immunity are still in their infancy (5).

Comprehensive epidemiological data on S. aureus has identified several distinct clades, each characterized by unique disease pathology and virulence factors. Among these are USA200 and USA300 strains, which are commonly referred to as hospital-acquired (HA) and community-acquired (CA) MRSA, respectively. Generally, HA-MRSA strains are robust formers of staphylococcal biofilm and demonstrate wider antimicrobial resistance profiles, and some clades (e.g., CC30) exhibit lower levels of secreted bacterial toxins (6). These strains are often associated with orthopedic and medical device-related infections. Conversely, CA-MRSA strains secrete comparatively higher levels of bacterial toxin, including Panton-Valentine leukocidin (PVL) and the pore-forming cytolytic alpha-toxin (1). These strains have been associated with skin infection outbreaks (e.g., in prisons, care facilities, locker rooms), and it is believed that high levels of toxin and others secreted factors allow for efficient skin-skin and skin-fomite transfer. Toxin expression in S. aureus has been linked to multiple bacterial sensory regulators, but perhaps the best studied is the *agr* (accessory gene regulator) quorum sensing system (7).

The *agr* system is the product of the RNAII transcript, consisting of an operon composed of four genes (*agrABCD*) (8). AgrD is the signal peptide, which gets secreted across the bacterial membrane into the extracellular space and modified to its mature form, autoinducing peptide 2 (AIP-2), via translocation through AgrB. AIP-2 is sensed by the cell surface-associated AgrC receptor that induces phosphorylation of AgrA. Activated AgrA can then bind again to the P2 promoter to increase RNAII transcription, completing a positive feedback cycle that is increased in a cell-density dependent fashion (i.e., quorum sensing). However, activated AgrA can also bind to the P3 promoter to drive expression of the RNAIII transcript, which directly encodes delta-toxin and a posttranscriptional regulatory RNA. Expression of RNAIII decreases transcription of *rot* (repressor of toxin), thereby increasing exotoxin secretion (9). Both alpha- and delta-toxin are well-characterized staphylococcal virulence factors, mediating a variety of pathological effects, including hemolytic activity, dermonecrosis, inflammasome activation, abscess formation, leukocyte oxidative burst, and reduced macrophage phagocytic killing (10–14). Toxin production by S. aureus is crucial for systemic disease, as high titers of antibody to staphylococcal exotoxins (including alpha-toxin) directly correlates with improved survival rates during clinical S. aureus sepsis (15).

A series of studies by Carlson demonstrated that peritoneal coinoculation of mice with C. albicans and S. aureus resulted in synergistic increases in mortality, while mice inoculated with each of these microbes alone efficiently cleared the infection (16–18). It was also observed that not all strains of S. aureus resulted in polymicrobial infectious synergism equally, with some decreasing the staphylococcal 50% lethal dose (LD_50_) by as much as 70,000-fold while others demonstrated only modest synergistic effects (2- to 3-fold) (16). It was proposed that the expression of specific staphylococcal toxins governed increased mortality, namely, alpha-toxin and delta-toxin. Unfortunately, lack of isogenic controls complicated data interpretation. Recently, our labs recapitulated these findings using a USA200 strain of S. aureus (NRS383) and identified that the host eicosanoid prostaglandin E_2_ (PGE_2_) is associated with disease severity and that pharmacologic blockade of PGE_2_ synthesis and PGE_2_ receptors 1 and 3 dramatically improves survival rate (19–21). Interestingly, staphylococcal exotoxins have been implicated in the activation of phospholipase A2 and subsequent prostaglandin release (22, 23). Furthermore, synergistic effects on mortality and PGE_2_ generation during coinfection with S. aureus are independent of the capacity to undergo fungal morphogenesis (the major virulence attribute of C. albicans) and are not limited to C. albicans, as various other *Candida* species (including C. dubliniensis, C. tropicalis, and C. krusei) also enhance morbidity and mortality during intra-abdominal infection (IAI) (19, 24).

Regarding the potential link to toxin expression and synergistic lethality with C. albicans, the objective of this study was to identify whether staphylococcal toxins may be required for lethal coinfection and/or whether toxin expression is elevated during polymicrobial IAI. In support of this, we show strong *in vitro* and *in vivo* evidence that staphylococcal alpha-toxin is necessary for robust infectious synergism and that C. albicans augments staphylococcal toxin production via engagement of the *agr* quorum sensing system.

## RESULTS

**FIG 1:**
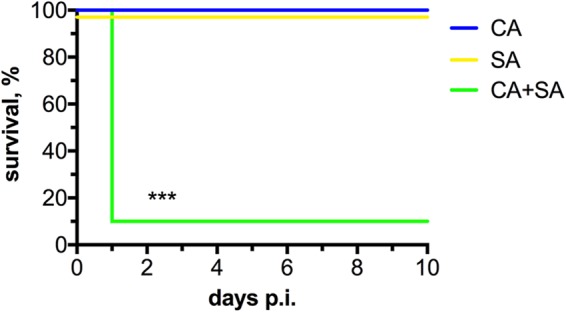
Coinfection with a USA300 strain leads to robust early synergistic mortality. Mice were inoculated i.p. with 1.75 × 10^7^ CFU of C. albicans (CA, blue), 8 × 10^7^ CFU of S. aureus (SA, yellow), or both pathogens simultaneously at these doses (CA+SA, green). Survival was monitored up to 10 days p.i. Data are derived from duplicate experiments of four mice per group and combined. Significance was assessed using a Wilcoxon log rank test. ***, *P* < 0.001.

Previous observations by Carlson revealed that S. aureus strains capable of producing various exotoxins demonstrate strong infectious synergism with C. albicans during IAI (16). S. aureus strain JE2 (derived from the USA300 isolate LAC) is a well characterized and robust producer of secreted toxins, with an intraperitoneal (i.p.) LD_50_ of 5 × 10^8^ CFU (25). Therefore, we administered a sublethal dose of JE2 to observe potential synergistic effects during coinfection. Intraperitoneal inoculation of Swiss-Webster mice with 8 × 10^7^ CFU of JE2 led to some morbidity by day 1 postinoculation (p.i.) (slightly ruffled fur), but ultimately these mice cleared the infection with no mortality (Fig. 1, SA, yellow). Similarly, infection with C. albicans alone led no observable morbidity and no mortality (Fig. 1, CA, blue). However, coinfection at this same dose with these microbes was striking (Fig. 1, CA+SA, green). Mice began exhibiting symptoms of morbidity around 12 h p.i. By approximately 16 to 20 h p.i., 80% of mice had succumbed to infection. Therefore, the rapid onset of morbidity and mortality in coinfected mice may be explained by the relatively high toxigenic activity of this S. aureus strain (among other strain-dependent differences). However, JE2 was nonlethal during monomicrobial infection. Thus, we hypothesized that coinfection with C. albicans may actually augment toxin expression.

**FIG 2:**
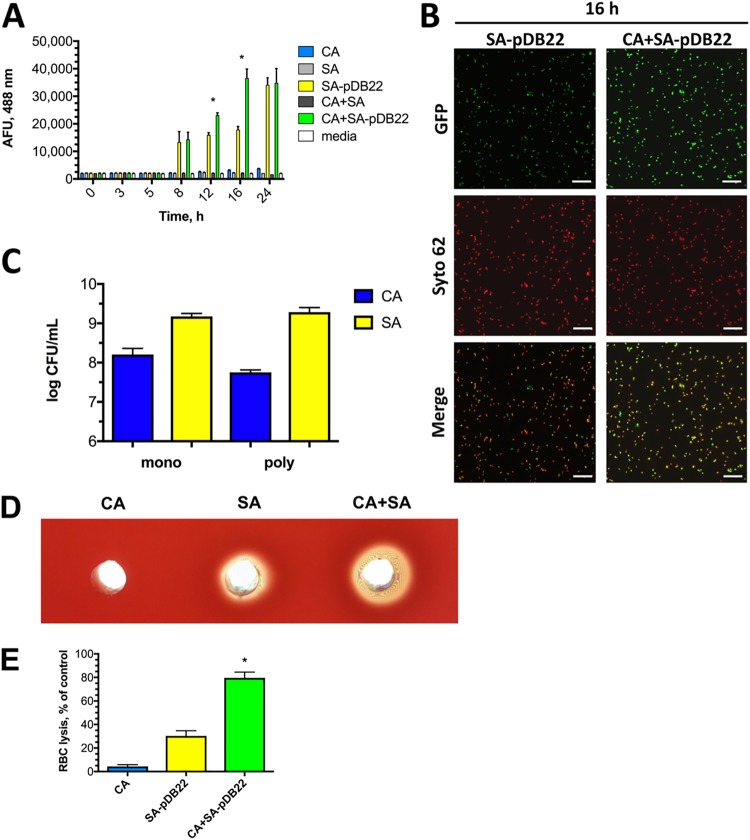
P3 reporter activity, a surrogate of staphylococcal *agr* activation, and red blood cell lysis are enhanced during coculture with C. albicans. (A) A P3-GFP reporter strain of S. aureus [S. aureus(pDB22)] was incubated alone (SA-pDB22) or with C. albicans (CA+SA-pDB22) over a 24-h time course. At each time interval, 100 μl of culture was removed in triplicate and added to a 96-well plate, and fluorescence was captured at 488 nm/515 nm. C. albicans only (CA) and S. aureus lacking pDB22 (SA) were also included as controls. Values represent the mean of results of triplicate experiments ± SEM. (B) Aliquots from reporter experiments were also visualized by fluorescence microscopy at 16 h by counterstaining cells with the DNA stain Syto62 and capturing GFP fluorescence with a GFP/Texas Red filter set. Images are representative of three independent repeats. (C) CFU levels (C. albicans, blue; S. aureus, yellow) of monomicrobial and polymicrobial cultures at 16 h of growth were assessed by microbiological plating on selective media. Counts were assessed for significance using a Mann-Whitney U test. (D) Hemolytic activity was assessed for monomicrobial (CA or SA) and polymicrobial (CA+SA) cultures by placing 20 μl of filtered culture supernatant into wells on a sheep’s blood agar plate. Images were captured after incubation at 37°C for 16 h and are representative of three independent repeats. (E) *agr* activity was quantitatively assessed by the capacity of supernatants from monomicrobial (C. albicans, blue; S. aureus, yellow) or polymicrobial cultures (green) to lyse rabbit erythrocytes. Values are expressed as the mean of results of triplicate experiments ± SEM. Significance was determined using a Mann-Whitney (CFU) or Student's *t* test (AFU count, RBC lysis). *, *P* < 0.05.

The staphylococcal *agr* quorum sensing system is tightly linked to toxin regulation. As part of a two-component signal transduction system, upon its activation, AgrA is phosphorylated and drives expression of RNAIII, the major toxin effector, by binding to the P3 promoter. We constructed an *agrA* reporter strain in JE2 based on plasmid pDB22 [S. aureus(pDB22)] with the P3 promoter fused to green fluorescent protein (GFP) for use as a proxy of toxin production (see Fig. S1 in the supplemental material). During planktonic culture in 0.6× Trypticase Soy Broth containing 0.2% glucose (TSB-g), GFP levels were absent at early time points, consistent with the fact that activation of the *agr* system is cell density dependent (Fig. 2A). At ∼8 h, reporter activities were noticeable in both monomicrobial and polymicrobial cultures harboring S. aureus(pDB22). However, at 12 and 16 h, reporter activities of polymicrobial cultures were significantly higher (as much as doubled) than those during monomicrobial growth, indicating augmented activation of the major signaling pathway controlling toxin production (Fig. 2A). To confirm these findings, aliquots from cultures were removed and imaged by fluorescence microscopy, revealing a qualitatively enhanced GFP signal in polymicrobial cultures in comparison to that in monomicrobial cultures (Fig. 2B). To address whether organism burden was drastically altered during polymicrobial growth at the 16 h time point, C. albicans, S. aureus, or the two combined were grown in TSB-g and plated on selective microbiological media. Enumeration revealed nearly identical C. albicans and S. aureus CFU levels between the monomicrobial and polymicrobial cultures (Fig. 2C). However, cell-free supernatants added to Trypticase Soy Agar (TSA)-blood revealed strikingly higher levels of hemolysis from polymicrobial cultures, suggesting enhanced levels or activity of hemolytic toxins during growth with C. albicans, consistent with GFP reporter results. Importantly, cell-free supernatants derived from C. albicans cultures were devoid of hemolytic activity (Fig. 2D). Augmented lysis of rabbit red blood cells (RBCs) (a cell type exquisitely sensitive to alpha-toxin) from polymicrobial cultures further confirmed this phenotype quantitatively (Fig. 2E) (26).

10.1128/mBio.00910-19.1 FIG S1 S. aureus strain JE2(pDB22) demonstrates GFP expression in a cell density-dependent manner, consistent with predicted *agr*/P3 promoter activity. Reporter plasmid pDB22 was isolated from S. aureus strain MN8 and transformed into strain JE2 to yield strain JE2(pDB22). The specificity of the GFP signal was assessed by inoculating both strains into TSB-g, incubating them at 37°C for 16 h, and imaging by fluorescence microscopy using a DIC/GFP filter set. Download FIG S1, TIF file, 0.7 MB. Copyright © 2019 Todd et al. 2019 Todd et al. This content is distributed under the terms of the Creative Commons Attribution 4.0 International license.

**FIG 3:**
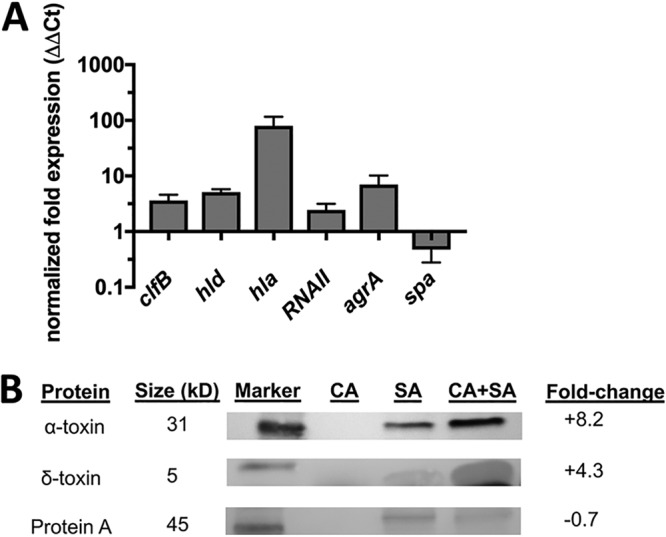
*agr*-associated genes and staphylococcal toxins are increased during polymicrobial growth with C. albicans. (A) Quantitative real-time PCR (qPCR) was performed to assess the expression of staphylococcal quorum sensing-related genes during monomicrobial versus polymicrobial culture. Values were calculated using the ΔΔ*C_T_* method and normalized to the reference gene *gyrB*. Data are expressed as normalized fold expression of polymicrobial versus monomicrobial cultures and the mean of results of three independent experiments. (B) Representative image (*n* = 3) of Western blot analysis performed using 40 μl of cell-free supernatants derived from C. albicans (CA) only, S. aureus (SA) only, or polymicrobial (CA+SA) cultures for alpha-toxin, delta-toxin, and protein A.

In order to determine whether downstream *agr*-regulated genes were also being increased during polymicrobial growth, we assessed the transcriptional responses of S. aureus or C. albicans in combination with S. aureus by quantitative real-time PCR (qPCR). By rupturing cells with lysostaphin (an enzyme that breaks down staphylococcal cell walls), we were able to selectively isolate S. aureus RNA so that the amounts of recovered staphylococcal nucleic acid between monomicrobial and polymicrobial cultures could be made equivalent. qPCR analysis revealed increases in *hld* (encoding delta-toxin, 5.1-fold), *hla* (alpha-toxin, 80-fold), RNAII (*agr* operon, 2.5-fold), *agrA* (quorum sensing regulator, 7-fold), and *spa* (surface protein A, –0.5-fold) (Fig. 3A). As RNAII*, hld*, *hla*, and *agrA* itself are downstream targets of *agr* activation, these results provide further evidence of augmented *agr* activity during growth with C. albicans. The exception to this gene list was the downregulation of *spa*. However, it has been well documented that *spa* is negatively correlated with *agr* activation, and thus its downregulation is in agreement with previous observations (27). Similar regulation of these quorum sensing effectors was also conserved at the protein level, as evidenced by Western blotting of cell-free supernatants (Fig. 3B). Phenol-soluble modulins (of which delta-toxin is a member) are small, ethanol-soluble peptides that prove somewhat difficult to resolve by electrophoresis (28, 29). Therefore, we utilized an ethanol precipitation step and subsequent drying to fractionate supernatants to improve toxin recovery, as described previously (28). Densitometric analysis demonstrated that alpha-toxin (37 kDa) was increased by 8.2-fold, delta-toxin (3 kDa) was increased by 4.3-fold, and protein A (56 kDa) was decreased by 0.7-fold, further explaining increased *in vitro* hemolytic activity. The protein expression levels were in line with transcriptional results, except that for alpha-toxin, which was lower than anticipated by qPCR and may indicate involvement of other mechanisms of posttranscriptional control (30).

**FIG 4:**
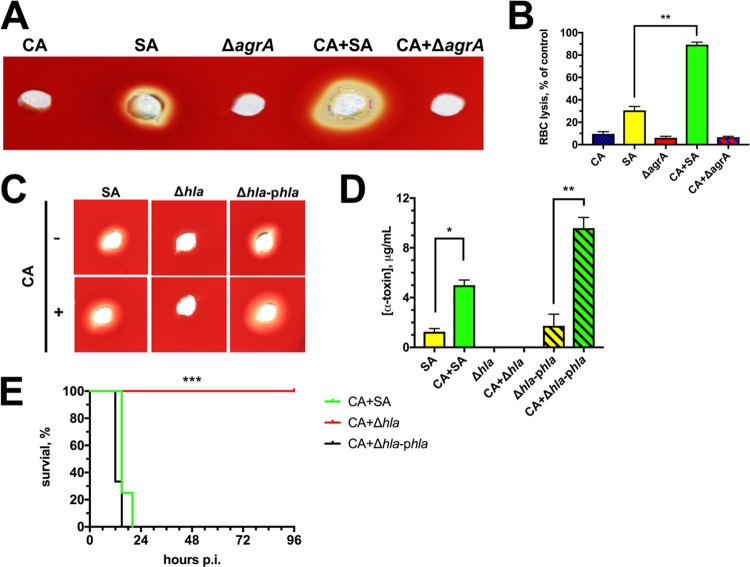
Increased S. aureus alpha-toxin during growth with C. albicans is *agr* dependent, and alpha-toxin is required for lethal infectious synergism during IAI. (A) Hemolytic activity of monomicrobial and polymicrobial cell-free supernatant (20 μl, 16 h time point) using S. aureus (SA) and a Δ*agr* deletion mutant was assessed using a sheep’s blood agar assay. A representative image of three independent repeats is shown. CA, C. albicans. (B) Lytic activity of culture supernatants against rabbit erythrocytes. Data are expressed as the mean (*n* = 3 experiments) ± SEM. **, *P* < 0.01. (C) An assay was performed as described for panel A using S. aureus, an Δ*hla* deletion mutant, and an *hla* complemented strain (Δ*hla*-p*hla*). A representative image of three independent repeats is shown. (D) Production of alpha-toxin as measured by ELISA during coculture of C. albicans (green bars) with wild-type S. aureus (yellow bar), a Δ*hla* deletion mutant (no data), or a Δ*hla*-p*hla* complemented strain (yellow hatched bar). Data are expressed as the mean ± SEM. *, *P* < 0.05, **, *P* < 0.01. (E) Mice were coinfected i.p. with C. albicans (CA) and wild-type S. aureus (green line), a Δ*hla* deletion mutant (red line), or a Δ*hla-*p*hla* complemented strain (black line) using established inocula. Mortality was monitored for up to 96 h p.i. Data are derived from duplicate experiments of four mice per group and combined. Significance was assessed using a Wilcoxon log rank test; ***, *P* < 0.001.

These findings revealed that *agr* activity was elevated during polymicrobial growth, although it was undetermined whether *agr* was required for augmented toxin secretion during coculture with C. albicans. Therefore, we assessed toxin secretion of a Δ*agrA* mutant during polymicrobial growth. Using the qualitative blood agar-based and quantitative RBC lysis assays, we confirmed that, indeed, enhanced toxin expression during coculture is *agrA* dependent, as growth with C. albicans was unable to rescue this *agr* defect (Fig. 4A and B). Given the strong upregulation of alpha-toxin observed during *in vitro* growth with C. albicans, we hypothesized that this toxin may drive synergistic lethality during coinfection. Use of an isogenic Δ*hla* strain revealed loss of lytic activity against sheep RBCs, indicating that the *in vitro* lytic phenotype is *hla* dependent (Fig. 4C). However, complementation of *hla* on a low-copy-number plasmid (Δ*hla*-p*hla*) restored the lytic phenotype to wild-type (WT) levels. Importantly, both the WT and complemented strains exhibited elevated RBC lysis during coculture with C. albicans (Fig. 4C). An alpha-toxin-specific enzyme-linked immunosorbent assay (ELISA) used to assess toxin levels in culture supernatants quantitatively confirmed these results (Fig. 4D). Alpha-toxin was required for early synergistic lethality during IAI, since coinfection with the Δ*hla* mutant was avirulent in comparison to coinfection with WT and Δ*hla*-p*hla* strains (Fig. 4E). As expected, coinfection with the Δ*agrA* mutant and monomicrobial infections were nonlethal over this same time course (data not shown).

**FIG 5:**
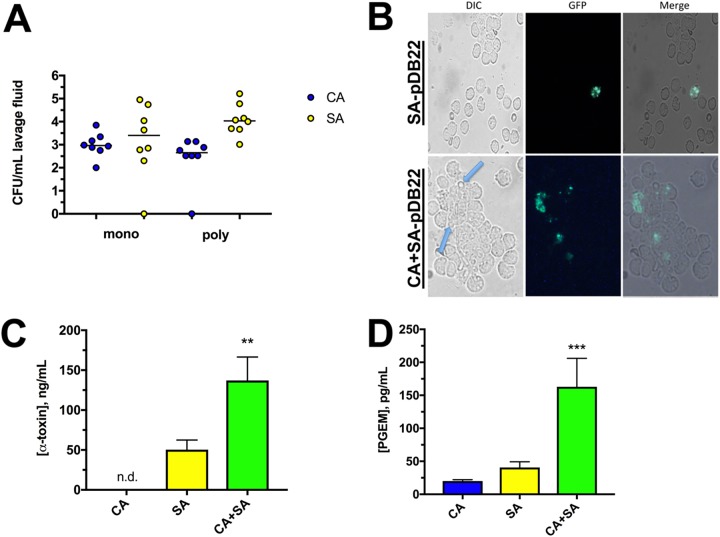
The S. aureus *agr* quorum sensing system is engaged during IAI, and polymicrobial infection leads to synergistic increases in PGE_2_ and alpha-toxin. (A) Mice (*n* = 4 per group) were inoculated with S. aureus (SA, yellow) or C. albicans (CA, blue) alone (mono) or with both pathogens simultaneously (poly) using established inocula. Mice were sacrificed 8 h p.i. and subjected to peritoneal lavage, and lavage fluid was plated on selective media. CFU counts are expressed as the median. (B) Mice were inoculated as described for panel A, except with strain S. aureus(pDB22), containing the GFP-P3 reporter (SA-pDB22) An aliquot of lavage fluid was assessed 8 h p.i. by fluorescence microscopy using DIC/GFP filter sets. Blue arrows depict hyphal filaments among peritoneal cells. Images are representative of at least 5 fields of view. (C) Levels of alpha-toxin present in peritoneal lavage fluid 8 h p.i. during monomicrobial (CA, blue; SA, yellow) and polymicrobial (CA+SA, green) infection as measured by ELISA. Data are cumulative of two independent repeats and expressed as the mean ± SEM. **, *P* < 0.01; n.d., not detected. (D) PGE_2_, as a measure of PGE metabolites (PGEM), present in the peritoneal lavage fluid 8 h p.i. as measured by competitive EIA. Data are cumulative of two independent repeats and expressed as the mean ± SEM. ***, *P* < 0.001. Synergistic significance was determined using a Mann-Whitney test (CFU) and Student's *t* test (alpha-toxin, PGEM).

We next wished to determine whether *agr* was engaged early during infection, prior to presentation of severe symptomatology. Mice were inoculated with either S. aureus, S. aureus(pDB22), C. albicans and S. aureus, or C. albicans and S. aureus(pDB22), sacrificed 8 h p.i., and subjected to peritoneal lavage. Analysis of the microbial burden in the lavage fluid revealed that similar to results in *in vitro* culture, the staphylococcal burdens were similar in monomicrobial and polymicrobial infections (Fig. 5A). Microscopic analysis of the lavage fluid revealed the presence of C. albicans and S. aureus, as well as a robust mononuclear infiltrate. The S. aureus(pDB22) strain demonstrated *agr* activity, as evidenced by GFP signal, but only sparingly and mostly in association with resident immune cells (Fig. 5B). In contrast, S. aureus(pDB22) coinoculated with C. albicans demonstrated a frequent GFP signal and when located near C. albicans (Fig. 5B). While this result is qualitative, it visually demonstrates that the *agr* system is indeed engaged in the murine peritoneal cavity. Importantly, at this early time point, there was a nearly 3-fold increase in alpha-toxin (Fig. 5C) and roughly a 4-fold increase in PGE_2_ (Fig. 5D) levels observed in the peritoneal lavage fluid of coinfected mice. Mice infected with C. albicans alone demonstrated modest PGE_2_ and no measurable alpha-toxin levels in the lavage fluid, demonstrating assay specificity.

**FIG 6:**
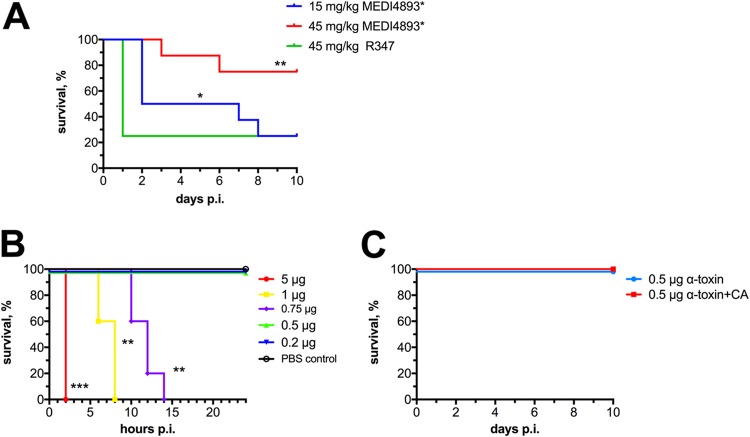
Alpha-toxin is necessary but not sufficient to drive infectious synergism during IAI. (A) Mice (*n* = 4 per group) were inoculated i.p. with 45 mg/kg human IgG1 isotype control antibody R347 (green) or an IgG1 antibody specific for alpha-toxin (MEDI4893*) at doses of 15 mg/kg (blue) or 45 mg/kg (red) 24 h prior to i.p. coinfection with C. albicans and S. aureus. Survival was monitored for up to 10 days p.i. Data are from duplicate experiments of four mice per group and were combined. Significance was assessed using a Wilcoxon log rank test. (B) A range of alpha-toxin doses were injected i.p. into groups of mice (*n* = 4) to determine lethality. (C) Swiss-Webster mice were inoculated i.p. with 0.5 μg of alpha-toxin, immediately followed by i.p. injection of either sham PBS (blue) or C. albicans. Data are representative of duplicate experiments with four mice per group, and data were combined. *, *P* < 0.05, **, *P* < 0.01, ***, *P* < 0.001.

Lastly, we wanted to determine whether a blockade of alpha-toxin was sufficient to elicit protection during polymicrobial IAI. Therefore, we passively immunized mice 24 h prior to coinfection with the anti-alpha-toxin-specific monoclonal IgG1 antibody (MAb) MEDI4893* at doses of 15 and 45 mg/kg or with the isotype control R347 (11). Mice receiving control antibody succumbed to coinfection within 1 day, similar to that previously observed in untreated mice (Fig. 6A). Mice receiving MEDI4893* at 15 mg/kg exhibited a slight delay in mortality (∼2 days p.i.). However, approximately 50% of these mice succumbed to infection by day 3 p.i., and this increased to 70% by day 8 p.i. Importantly, mice receiving MEDI4893* at a dose of 45 mg/kg exhibited no mortality until day 4 p.i. and an overall mortality rate of only 30% (Fig. 6A). Thus, passive administration of MEDI4893* improves survival during polymicrobial IAI. We next wanted to determine whether administration of alpha-toxin during infection with C. albicans was sufficient to cause synergistic lethality. First, a nonlethal i.p. dose of alpha-toxin (0.5 μg) was determined (Fig. 6B). However, coinoculation of mice with C. albicans and 0.5 μg alpha-toxin did not recapitulate the lethal synergy between C. albicans and S. aureus (Fig. 6C). Collectively, these results demonstrate that the staphylococcal regulator *agr* is robustly increased during fungal-bacterial coinfection and that alpha-toxin is necessary but not sufficient to mediate early lethal synergism with C. albicans.

## DISCUSSION

In nature, microorganisms rarely grow as single species; instead, most exist as polymicrobial communities characterized by mutualistic, parasitic, commensalistic, or antagonistic interactions. With the advent of high-throughput sequencing technologies, we have now begun to realize the tremendous microbial diversity that exists, not only in the environment but also within the human biome. Although we are rapidly amassing incredible amounts of data regarding microbiome composition, functional and mechanistic relationships between microbial consortia and the human host are only now being explored in depth. Furthermore, the boundaries of Koch’s postulates are being challenged, as some disease phenotypes are now realized to result from a complex set of microbial virulence mechanisms, partially governed by microbe-microbe interactions. One extreme example is “lethal synergism,” in which coinfection mediates the mortality of otherwise survivable infections with each microbe independently. This phenomenon has been previously reported in a variety of biological systems but is perhaps best characterized by fungal-bacterial IAI with C. albicans and S. aureus.

Our labs have explored the interaction between C. albicans and S. aureus extensively. Previous studies revealed that, indeed, C. albicans influenced global protein expression during growth with S. aureus, including the modulation of several putative virulence factors (31). It has also been determined that S. aureus preferentially binds the invasive hyphal form of C. albicans via the candidal adhesin Als3p and that this interaction allows for enhanced mortality and systemic dissemination from the oral cavity during oropharyngeal candidiasis (32–34). Furthermore, during biofilm growth, C. albicans confers high-level vancomycin resistance to S. aureus by forming a drug permeability barrier via elaboration of a carbohydrate-dense extracellular matrix (35, 36). These studies highlight the highly dynamic relationship between these microbes.

Data in this study build off recent efforts by our labs seeking to define the mechanism by which C. albicans augments S. aureus infection during polymicrobial IAI, as observed by Carlson many years earlier. Initial studies determined that host inflammation, including the eicosanoid PGE_2_ and cytokine interleukin-6 (IL-6), was synergistically exacerbated in mice challenged with the two microbes simultaneously compared to that of monomicrobial infection and correlated with a lethal outcome (24). Furthermore, protection was mediated by anti-inflammatory administration and subsequent PGE_2_ blockade (20, 21). Using various transcriptional regulator mutants, lethal infection was surprisingly independent of C. albicans morphological transition, as other experimental C. albicans disease models are almost universally dependent on morphogenetic regulation (19). This observation was further confirmed by the fact that various other *Candida* species mediate synergistic lethality independent of the capacity to form true hyphae during IAI (24). Importantly, all of these studies were conducted using S. aureus strain NRS383 (USA200), which is grouped into the CC30 clonal complex (37). While synergistic lethality was achieved during coinfection with this strain, kinetics (∼day 3 p.i.) were modest compared to that of infection with JE2 (<day 1 p.i.) in this study. Interestingly, CC30 strains are documented to have very low to no production of alpha-toxin under *in vitro* conditions, in some cases due to a premature stop codon within *hla* (38). NRS383 exhibits a weak zone of hemolysis on blood agar, further emphasizing the importance of staphylococcal alpha-toxin in driving lethal synergism with C. albicans.

The major staphylococcal regulator of alpha-toxin production is indeed governed by *agr*-dependent signaling. However, several reports suggest contributions of *agr*-independent mechanisms regulated via the *sarA* and *saeRS* networks (39). In support of this, clinical isolates that are *agr* negative have been observed to activate *hla* transcription *in vivo*. Similarly, deletion of *sae* causes loss of *hla* transcription, despite an intact *agr* regulon (40). Results from our previous study of polymicrobial IAI demonstrated that S. aureus disseminates to distant tissues (including the brain) quickly and, at later time points, reaches greater numbers during polymicrobial infection than during monomicrobial infection (19). Thus, it is possible that delayed activation of these alternative alpha-toxin regulators at sites of dissemination *in vivo* may explain lethality with strains exhibiting low hemolytic activity *in vitro*. Given that a relatively large fraction (15 to 60%) of hospital-acquired MRSA strains are *agr* defective, it will be interesting to delineate the role of these alternative alpha-toxin regulators in driving synergistic lethality and their potential activation during growth with C. albicans (41).

Experiments utilizing the isogenic Δ*hla* strain during coinfection demonstrated a clear requirement for alpha-toxin to mediate infectious synergism. These findings were corroborated by a study by Rauch et al., in which loss of *hla* during high-dose monomicrobial peritoneal challenge with S. aureus strain Newman dramatically improved survival rates over that of wild-type infection (25). Moreover, blockade of alpha-toxin using the investigational neutralizing antibody MEDI4893* further confirmed the importance of this virulence determinant in driving mortality during IAI. MEDI4893* exerts its effects by not only inhibiting engagement of alpha-toxin with its host receptor ADAM10 but also by preventing oligomerization of the mature heptamer required for membrane pore formation and lytic activity (42). Aside from its clear protective effects demonstrated against polymicrobial IAI in this study, MEDI4893* has also demonstrated efficacy in both immunocompetent and immunocompromised animal models, including models of necrotizing pneumonia, dermonecrosis, and sepsis (11, 43–45). Although prophylactic treatment with MEDI4893* did not confer 100% survival in this study, it did significantly delay mortality to 2 (15 mg/kg) and 4 (45 mg/kg) days p.i., respectively, which could allow for an extended therapeutic window for antimicrobial administration. Although beyond the scope of this work, future studies to determine whether MEDI4893* can be given therapeutically to confer protection postinfection are warranted. In any case, passive immunization against alpha-toxin is an intriguing possibility for those at elevated risk of IAI, including abdominal surgery, peritoneal dialysis, or tertiary peritonitis patients.

Cohen et al. recently demonstrated that alpha-toxin is necessary and sufficient for S. aureus lethal synergy during lung coinfection with Gram-negative opportunists (46). However, it is still unclear whether alpha-toxin is sufficient to mediate lethal synergism during C. albicans infection or if other components of S. aureus are also required. Carlson had previously observed that inoculation of C. albicans with cell-free spent culture supernatants of S. aureus could induce synergistic lethality, suggesting that staphylococcal secreted products (e.g., toxins) were perhaps implicated (18). The potency of alpha-toxin is impressive, with the LD_50_ being reported as ∼1 μg via systemic injection or intranasal challenge; an intradermal dose of 10 μg is sufficient to induce large skin lesions (11, 13, 47). Despite initial signs of morbidity, inoculation of mice with C. albicans, followed by a sublethal dose of purified alpha-toxin, was unable to recapitulate synergistic mortality (Fig. 6). There are several reasons why this may be the case. It is possible that a sublethal bolus dose of toxin is not sufficient to potentiate the effects with C. albicans. It is also equally likely that sustained expression of S. aureus toxin during infection is required to augment coinfection. Alternatively, alpha-toxin may only potentiate host- or microbe-derived signals present during infection with intact fungal or bacterial pathogens or may be required to be present with C. albicans at the immunological synapse. Additionally, Carlson’s observations may be explained by the presence of multiple toxins or shed cell wall material present in the culture supernatant that was not completely inactivated by heating. These soluble factors may maintain proinflammatory activity to elicit robust PGE_2_ and cytokine production during coinfection by stimulating pattern recognition receptors (PRRs) and serving as danger-associated molecular patterns (DAMPs).

This study describes the novel finding of a human-pathogenic fungus engaging the virulence of a bacterial pathogen with dire consequences for the host. That said, cross-species and cross-domain quorum sensing communication has been observed in several other reports. For example, Bamford et al. demonstrated that the diffusible chemical signal AI-2, derived from the *luxS* gene of the ubiquitous oral commensal Streptococcus gordonii, enhances C. albicans hyphal formation during polymicrobial biofilm growth (48). Perhaps one of the best-studied interactions occurs between C. albicans and Pseudomonas aeruginosa, whereby the bacterial quorum sensing molecule 3-oxo-C12 homoserine lactone inhibits the fungal yeast-to-hypha transition (49). P. aeruginosa also attaches to the fungal surface, secreting a variety of fungicidal substances (phenazine, pyocyanin, etc.) (50). In response, C. albicans secretes its own quorum sensing compound, farnesol, which has antibacterial activity (51). Therefore, it is not unreasonable to speculate that secreted fungal quorum sensing molecules (or otherwise) may elicit toxigenic responses by S. aureus via quorum sensing induction. Indeed, at supraphysiologic concentrations, farnesol does exhibit antistaphylococcal effects (52). However, an apparent lack of an antagonistic relationship between these microbes during coculture, as evidenced by previous imaging studies, further clouds this hypothesis (31).

It is possible that cell-cell contact, and not necessarily a secreted factor, mediates the toxigenic response in S. aureus. Previous studies from our labs have demonstrated that the major adhesin responsible for C. albicans-S. aureus interactions is the candidal protein Als3p (33). This fact, coupled with the ability of several transcriptional regulator mutants of C. albicans incapable of hypha formation to still promote lethal synergism during IAI, suggests an alternative mechanism, as these strains would also be predicted to not express hypha-associated Als3p (19, 24). Furthermore, coinfection with a Δ/Δ*als3* mutant of C. albicans still exhibited synergistic lethality with JE2 during experimental IAI (unpublished studies). That said, maximal engagement of a soluble factor to its potential target *in vivo* may occur if both microbes maintain intimate contact. We also cannot rule out the possibility that an environmental factor or host component mediates enhanced toxin secretion *in vivo*. It is well known that *agr* is sensitive to several environmental stimuli, including pH; the pH of the abdominal cavity during C. albicans peritonitis is slightly alkaline (53, 54). Thus, it is possible that anatomical niches within the abdomen buffer the acidic end products of staphylococcal metabolism, thereby maintaining neutral pH levels optimal for sustained *agr* activation. C. albicans infection may also generate host or fungal by-products that S. aureus can use as metabolic substrates or signaling molecules to upregulate toxin expression.

Collectively, these results demonstrate that C. albicans augments S. aureus virulence via an *agr*- and alpha-toxin-dependent mechanism during polymicrobial IAI, resulting in devastating consequences for the host. These findings are important, given the frequency with which these microbes are coisolated from biologic (e.g., vagina, gastrointestinal tract, skin, oropharynx) and artificial (e.g., indwelling catheters) sites during colonization or infection. Furthermore, systemic disease caused by *Candida* species is associated with mortality rates exceeding 40% even with appropriate therapy (55). Given our observations, it is possible that these unacceptably high mortality rates may not only result from fungal infection alone but may also be engaging virulence mechanisms in otherwise commensal microbes or coinfecting pathogens (56–58). Novel strategies to inhibit fungal-bacterial quorum sensing cross talk or its downstream effectors may be warranted to better manage IAI, including the use of an antibody-mediated vaccine against staphylococcal alpha-toxin in at-risk patient populations to limit the incidence of severe sepsis.

## MATERIALS AND METHODS

### Ethics statement.

The animals used in this study were housed in AAALAC-approved facilities located at the University of Tennessee Health Sciences Center (UTHSC) in the Regional Biocontainment Laboratory. The UTHSC Animal Care and Use Committee Laboratory Animal Care Unit (LACU) approved all animal usage and protocols (protocol number 18-060). Mice were given standard rodent chow and water *ad libitum*. Mice were monitored daily for signs of distress, including noticeable weight loss and lethargy. UTHSC LACU uses the Public Health Policy on Humane Care and Use of Laboratory Animals (PHS) and the *Guide for the Care and Use of Laboratory Animals* as a basis for establishing and maintaining an institutional program for activities involving animals. To ensure high standards for animal welfare, UTHSC LACU remains compliant with all applicable provisions of the Animal Welfare Act (AWAR), guidance from the Office of Laboratory Animal Welfare (OLAW), and the American Veterinary Medical Association Guidelines on Euthanasia.

### Strains and growth conditions.

C. albicans strains SC5314 (wild type, reference isolate) was used for all experiments. The following S. aureus strains obtained from the Biodefense and Emerging Infectious (BEI) Research Resources repository were used: JE2 (WT; USA300 derived from strain LAC but cured of erythromycin resistance and cryptic plasmids), NE1532 (Δ*agrA*), and NE1354 (Δ*hla*) (59). The S. aureus reporter strain harboring plasmid pDB22 encoding erythromycin resistance and a P3 promoter fused to GFP_mut2_ was a kind gift from Pete Greenberg (University of Washington) (60). Using standard techniques, plasmid pDB22 was isolated from MN8-pDB22 and transformed into strain JE2 by electroporation to yield S. aureus(pDB22) (61). Using fluorescence microscopy, it was confirmed that S. aureus(pDB22) exhibited robust GFP expression during stationary growth, consistent with *agr* activation (see Fig. S1 in the supplemental material). All strains were maintained as 20% glycerol stocks and stored at −80°C.

Unless specifically noted otherwise, C. albicans was streaked onto Yeast Peptone Dextrose (YPD) agar. Isolated colonies were selected and inoculated into liquid YPD medium and grown overnight at 30°C with shaking at 200 rpm. S. aureus was streaked onto Trypticase Soy Agar (TSA) with antibiotic selection as required. Isolated colonies were selected and inoculated into liquid Trypticase Soy Broth (TSB) medium and grown overnight at 37°C with shaking at 200 rpm. The following day, cultures were diluted 1:100 in fresh TSB and returned to the shaking 37°C incubator for 3 h until cultures reached the logarithmic phase of growth.

### Construction of an *hla*-complemented strain.

In order to complement alpha-toxin expression, primers (restriction enzyme sites indicated by underlined text) hlaF-HindIII (5′-GTAAAGCTTCATACGATACTTTTTCGTTATCTATTAG) and hlaR-BamHI (5′-CGGGGATCCCAGTATAAAAATTAGCCGAAAAACATCATTTCTG) were used to PCR amplify genomic DNA isolated from strain JE2 to generate a fragment containing the entire *hla* open reading frame, ∼500 bp of the 5′untranslated region (UTR), and ∼100 bp of the 3′UTR. Vector pSK5630 (a low-copy-number plasmid, ∼5 per cell) and the *hla* PCR product were digested with HindIII and BamHI and ligated to yield plasmid pSK-hla. The *dcm*-deficient Escherichia coli strain IM08B was made chemically competent and used as a propagation host for pSK-hla after standard heat shock transformation and selection on Luria-Bertani (LB) agar containing ampicillin (50 μg/ml) to yield strain IM08B-phla (62). pSK-hla was isolated from selective overnight cultures of IM08B-phla using a miniprep procedure according to the manufacturer’s protocol (GeneJet; ThermoFisher) and verified by restriction digestion with HindIII and BamHI, followed by Sanger sequencing (UTHSC Molecular Resource Center). Using a vendor-optimized protocol for staphylococcal electroporation (Gene Pulser Xcell; Bio-Rad), pSK-hla was transformed directly into strain NE1354 (Δ*hla* background) and plated on brain heart infusion agar containing chloramphenicol (10 μg/ml) to yield the Δ*hla*-p*hla* strain. Individual colonies were replica plated onto TSA containing 5% sheep’s blood and chloramphenicol (10 μg/ml) to screen for those with a hemolytic phenotype. Plasmids were isolated from hemolysis-positive colonies by a miniprep procedure using an S. aureus-specific manufacturer-supplied protocol (Qiaprep; Qiagen) and verified by PCR amplification using primers pSK5630-F (5′-ACGATGCGATTGGGATATATCAACG) and hlaDETR (5′-GTGTTGTTGTTACTGAGCTGACTATACG).

### Murine model of IAI.

Intraperitoneal inoculations were conducted as described previously (19, 20, 24). In most experiments, groups (*n* = 4) of 6-week-old Swiss Webster mice were injected intraperitoneally (i.p.) using a 27-gauge 0.5-in needle with 1.75 × 10^7^ CFU of C. albicans, 8 × 10^7^ CFU of S. aureus, or both 1.75 × 10^7^ and 8 × 10^7^ CFU of the respective microbes simultaneously. Inocula were prepared in a final volume of 0.2 ml pyrogen-free phosphate-buffered saline (PBS). After inoculation, mice were observed up to 10 days for morbidity (hunched posture, inactivity, ruffled fur) and mortality. Mice that exhibited severe morbidity were humanely sacrificed and tallied as a lethal outcome. In some experiments, mice were sacrificed 8 h p.i. prior to severe morbidity. Peritoneal cavities were lavaged by injection of 2 ml of sterile PBS containing 1× protease inhibitors (cOmplete; Roche), followed by gentle massaging of the peritoneal cavity. Peritoneal lavage fluid was then removed using a pipette inserted into a small incision in the abdominal cavity. Animal experiments were repeated in duplicate, and results were combined.

### Passive immunization with anti-alpha-toxin monoclonal antibody.

Groups of mice (*n* = 4) were inoculated i.p. with 200 μl of the alpha-toxin-specific IgG1 neutralizing antibody MEDI4893* (15 mg/kg, 45 mg/kg) or human IgG1 isotype control R347 (45 mg/kg) prepared in sterile PBS 24 h prior to coinfection with C. albicans and S. aureus. Mice were monitored for up to 10 days p.i. for morbidity and mortality. Animal experiments were repeated in duplicate, and results were combined.

### Intraperitoneal delivery of alpha-toxin.

Groups of mice (*n* = 4) were inoculated i.p. with 0.2, 0.5, 0.75, 1, or 5 μg of purified native alpha-toxin in 0.1 ml of PBS alone or sequentially with 1.75 × 10^7^ CFU of C. albicans in 0.1 ml PBS (13). Mice inoculated with alpha-toxin only received a sham injection of 0.1 ml PBS. Mice were monitored for morbidity and mortality up to 10 days p.i. Data are representative of at least two independent repeats.

### CFU analysis.

Microbial burdens were enumerated by serial dilution plating of peritoneal lavage fluid and culture media onto YPD containing 20 μg/ml ampicillin and 2 μg/ml vancomycin (for C. albicans enumeration) and TSA containing 20 μg/ml ampicillin and 2.5 μg/ml amphotericin B (for S. aureus enumeration) via the drop-plate method (63). The plates were incubated overnight at 37°C, and the microbial burden was enumerated and expressed as the number of CFU per ml. CFU values are representative of at least two independent repeats (*n* = 4 mice per group) and are represented as the median (±standard deviation [SD]).

### Prostaglandin quantitation.

Recovered lavage fluid was centrifuged at 500 × *g* for 5 min, and the supernatant was transferred to clean microcentrifuge tubes. PGE_2_ is rapidly converted to its 13,14-dihydro-15-keto metabolite *in vivo*, so the colorimetric prostaglandin E metabolite competitive enzyme immunoassay (EIA) was used to measure PGE_2_ as a function of its breakdown product, according to the manufacturer’s directions (Cayman Chemicals, Ann Arbor, MI). This assay is highly sensitive and detects as little as 2 pg/ml of PGE metabolites.

### S. aureus alpha-toxin quantitation by ELISA.

Wells of a polystyrene 96-well microtiter plate were coated with 50 μl of 0.1 μg/ml anti-alpha-toxin antibody MEDI4893* (diluted in coating buffer [0.2 M carbonate/bicarbonate buffer]) and incubated overnight at 4°C. The plates were washed sequentially with PBS-Tween 20 (PBS-T) and blocked with SuperBlock (Pierce) for 1 h at room temperature. After washing with PBS-T, 50 μl of diluted peritoneal lavage fluid or diluted filter-sterilized culture supernatants in PBS was added. Additionally, serial dilutions of native alpha-toxin were included as the standard curve. The plate was then incubated for 1 h at room temperature with shaking (600 rpm). After washing with PBS-T, 50 μl of affinity-purified rabbit polyclonal anti-alpha-toxin antibody (2 μg/ml) was added and incubated as described above. After washing, 50 μl of a 1:10,000 dilution of AffiniPure horseradish peroxidase (HRP)-coupled goat anti-rabbit IgG detection antibody (Jackson ImmunoResearch) was added and incubated for 1 h. The plates were washed extensively with PBS-T, and then 100 μl of 3,3’,5,5’-Tetramethylbenzidine (TMB) substrate was added for 10 min, followed immediately by the addition of 100 μl of ELISA stop solution (0.2 M H_2_SO4). Wells were read at 450 nm using a plate reader (Synergy; BioTek), and experimental values were extrapolated to the standard curve. Culture supernatants and peritoneal lavage fluid generated from the Δ*hla* strain were blank subtracted to account for any potential nonspecific antibody binding to shed protein A. Results were repeated in duplicate (*n* = 4 mice per group), and data were combined and expressed as the mean ± standard error of the mean (SEM).

### Agar plate assay for toxin activity.

Colonies of S. aureus (JE2 and Δ*agrA* and Δ*hla* strains) and C. albicans were grown overnight as described above, and cell densities were adjusted to 1 × 10^7^ CFU/ml by counting on a hemocytometer. A 1:100 dilution (50 μl) was made into 5 ml of 0.6× TSB containing 0.2% glucose (TSB-g) (a medium previously shown to support good growth of both C. albicans and S. aureus) to establish the following groups: S. aureus alone, C. albicans alone, or C. albicans plus S. aureus (64, 65). Monomicrobial cultures received an additional 50 μl of sterile PBS. Tubes were placed in a 37°C incubator with shaking at 200 rpm. Aliquots were removed at several time points, centrifuged at 5,000 rpm to remove cellular debris, and passed through a 0.2-μm syringe filter. Wells were aseptically formed in TSA agar containing 5% sheep’s blood using a cut sterile pipette tip. Sterile culture supernatant (20 μl) was added to each well, and the plate was incubated at 37°C for up to 24 h. Plates were imaged using a digital scanner, and the results are representative of at least three independent repeats.

### RBC hemolytic assay.

An aliquot (1 ml) of rabbit blood (Lampire Biologicals) was centrifuged at 1,000 × *g* for 5 min to pellet red blood cells (RBCs). Packed RBCs were washed two more times by centrifugation and resuspended in 1 ml of PBS, and a 4% (vol/vol) dilution of RBCs was made. Isolated cell-free supernatants (as described above) were serially diluted in sterile TSB-g, and 200 μl was added to wells of a microtiter plate. To this, 50 μl of RBCs was added, and the plate was incubated at 37°C for 1 h with gentle shaking. Plates were centrifuged at 1,000 × *g* for 5 min to pellet unlysed RBCs, supernatants were transferred to a fresh microtiter plate, and the optical density at 405 nm (OD_405_) was collected as a measure of hemoglobin release. Positive (1% Triton X-100) and negative (sterile TSB-g) controls were included. Experiments were repeated in triplicate, and values were normalized as the percentage of results for the positive control and expressed as the mean ± SEM.

### *agrA*-GFP reporter assay.

The culture setup for the *agrA*-GFP reporter assay was similar to that of the agar plate assay described above, except that the S. aureus(pDB22) strain was also included and 10 μg/ml of erythromycin was included for plasmid maintenance. Aliquots of cultures (100 μl) were removed at various time points postinoculation in TSB-g and placed into wells of a black microtiter plate in triplicate. Fluorescence (488 nm excitation, 515 nm emission) was captured on a plate reader (Synergy; BioTek). Experiments were repeated in triplicate, and results are expressed as mean arbitrary fluorescence units (AFUs) ± SEM.

### Fluorescence microscopy.

Aliquots of cells from the reporter assay were stained with the fluorescent DNA stain Syto62 per the manufacturer’s protocol (Invitrogen) and placed onto coverslipped glass slides. Images were captured on an Olympus FV1000 confocal microscope using GFP and Texas Red filter sets. Samples of peritoneal lavage fluid were placed onto glass slides and imaged using differential interference contrast (DIC) and GFP filter sets. Images are representative of at least three independent repeats.

### RNA isolation for quantitative real-time PCR (qPCR).

Cultures were set up as described for the agar plate assay. RNA from S. aureus was selectively isolated as described previously, with some modifications (66). Briefly, cells were removed from the incubator and an equal volume of ice-cold 50% acetone–50% ethanol was added to prevent RNA degradation. Cells were centrifuged twice at 4,000 rpm and resuspended in 1 ml of diethyl pyrocarbonate (DEPC)-treated water. Cells were transferred to Eppendorf tubes and centrifuged, and 5 μl of lysostaphin was added (10 mg/ml). The pellet was vortexed vigorously, resuspended in 200 μl of DEPC-treated water, and incubated in a 37°C water bath for 45 min. To this, 2.5 μl of proteinase K (Qiagen) was added and incubated for an additional 15 min. After incubation, 0.2 volumes of 10% SDS was added, followed by an equal volume of a 25:24:1 phenol-chloroform-isoamyl alcohol mixture at pH 4 and a small amount of 0.1-mm-diameter zirconia beads. The tubes were vortexed vigorously for 2 min, followed by 1 min of incubation on ice; this was repeated three times in total. RNA was extracted by the hot phenol-chloroform method (5:1, pH 4), precipitated with 3 M sodium acetate, and washed with 70% ethanol. The pellet was air-dried and resuspended in 25 μl DEPC-treated water. Concentrations were assessed spectrophotometrically (*A*_260_/*A*_280_ ratio), and RNA integrity was verified by running ∼1 μg of RNA on a 1.4% Tris-borate-EDTA (TBE) agarose gel. Analysis of 16S and 23S (but lack of 18S and 28S) rRNA bands confirmed that staphylococcal RNA from polymicrobial cultures was selectively extracted by this procedure.

### qPCR for staphylococcal genes.

Trace amounts of contaminating DNA were removed from RNA samples (1 μg) by treatment with RNase-free DNase I per the manufacturer’s protocol (Thermo Fisher). RNA was reverse transcribed using the RevertAid first-strand kit and primed with random hexamers per the manufacturer’s protocol (Thermo Fisher). cDNA (100 ng) was amplified using gene-specific primers (Sigma) and the Maxima Sybr green 2× PCR mastermix per the manufacturer’s protocol (Thermo Fisher). Amplification and fluorescence measurement were conducted using the 7500 real-time PCR system platform (Applied Biosystems). Expression levels of target genes in polymicrobial cultures were compared to those in monomicrobial cultures and normalized to the staphylococcal reference gene *gyrB* using the ΔΔ*C_T_* method as described previously (67). RNA was extracted from triplicate experiments, and ΔΔ*C_T_* values were calculated, averaged, and expressed as the mean ± SD.

### Western blotting for staphylococcal proteins.

Cell-free supernatants were separated into ethanol-soluble and -insoluble fractions by treatment with 80% (vol/vol) final concentration ice-cold ethanol for 1 h at −80°C. Samples were centrifuged at 3,000 × *g* for 15 min. The ethanol-soluble fraction was transferred to a clean tube, and both fractions were placed in an N-EVAP nitrogen evaporator (Organomation) set at 75°C and 10-mm flow. Upon reaching near-dryness, samples were resuspended in 0.5 ml of ultrapure water, and total protein content was assessed by the bicinchoninic acid (BCA) assay (Pierce). Equivalent volumes (40 μl) of concentrated supernatants were diluted in 6× Laemmli sample buffer, boiled, and separated by SDS-PAGE in duplicate. We chose not to normalize results to total protein, as the polymicrobial culture would have elevated levels of protein (both C. albicans and S. aureus) and may bias interpretation when it was compared to monomicrobial cultures. Ethanol-soluble fractions were assessed using Tris-Tricine electrophoresis, and ethanol-insoluble fractions were assessed using a Tris-glycine system. An equivalent protein load was confirmed by staining one set of gels with BioSafe Coomassie (data not shown), while the other gel was transferred to nitrocellulose membranes and blocked in 5% powdered milk PBS-T for antibody probing as follows. To detect alpha-toxin, a 1:10,000 dilution of primary rabbit anti-alpha-toxin (Sigma; S7531) and 1:50,000 dilution of HRP-coupled anti-rabbit IgG antibodies were used. To detect delta-toxin, a 1:1,000 dilution of primary rabbit anti-delta-toxin (LSBio, LS-C156026) and a 1:50,000 dilution of HRP-coupled anti-rabbit IgG antibodies were used. To detect protein A, a 1:2,000 dilution of mouse anti-protein A (Abcam; ab181627) and a 1:50,000 dilution of goat anti-mouse IgG (Fab specific) were used. Primary antibodies were incubated overnight at 4°C in blocking buffer with gentle rocking. Secondary antibodies were incubated for 1 h at room temperature with gentle rocking. Gels were washed at least three times for 5 min with PBS-T between incubation steps. Signal was detected using the SuperSignal chemiluminescent substrate kit (Pierce) per the manufacturer’s protocol, and exposure images were captured on the ChemiDoc XRS system (Bio-Rad). Densitometry of the resulting bands was calculated using ImageJ software (NIH). Blots are representative of three independent repeats.

### Statistical analyses.

A two-tailed Mann-Whitney test was used to compare CFU values between polymicrobial and monomicrobial groups. A two-tailed unpaired Student’s *t* test was used to compare AFU and RBC lysis values for polymicrobial versus monomicrobial groups. A Wilcoxon log rank test was used to determine the significance of mortality plotted in Kaplan-Meier curves. Statistical analyses were performed using GraphPad Prism. All graphs were constructed using GraphPad Prism. Figures were composed using MS Powerpoint and rendered for publication with Adobe Photoshop.
