# Supplementary figures and images for: Candida albicans Augments Staphylococcus aureus Virulence by Engaging the Staphylococcal agr Quorum Sensing System
Source: mBio. 2019 Jun 4;10(3):e00910-19. doi: 10.1128/mBio.00910-19 (PMC6550526; doi:10.1128/mBio.00910-19)

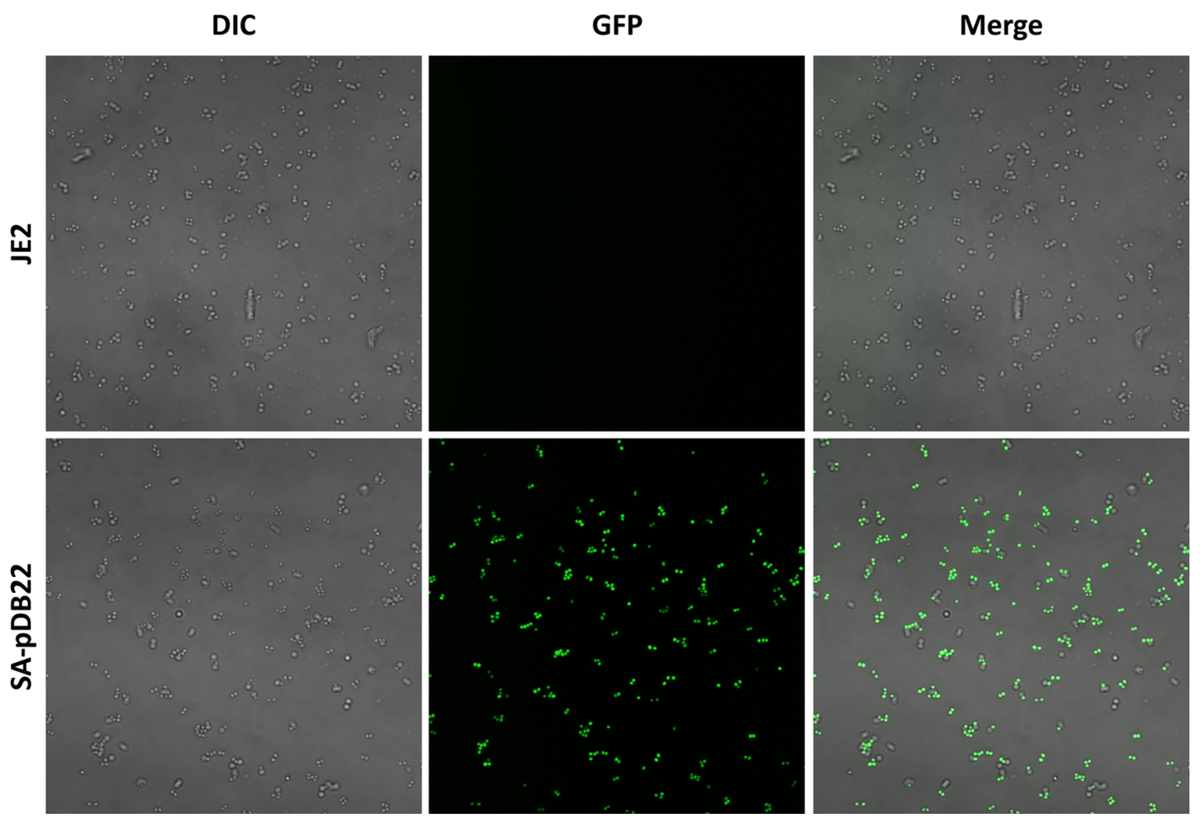

Supplement: FIG S1 [file mBio.00910-19-sf001.tif]
